# Laboratory-Reported Normal Value Ranges Should Not Be Used to Diagnose Periprosthetic Joint Infection

**DOI:** 10.7759/cureus.28258

**Published:** 2022-08-22

**Authors:** Salvador A Forte, Joseph A D'Alonzo, Zachary Wells, Brett Levine, Stephen Sizer, Carl Deirmengian

**Affiliations:** 1 Orthopedic Surgery, Philadelphia College of Osteopathic Medicine, Philadelphia, USA; 2 Orthopedic Surgery, Rush University Medical Center, Chicago, USA; 3 Orthopedic Surgery, Thomas Jefferson University, Philadelphia, USA; 4 Orthopedic Surgery, The Rothman Orthopedic Institute, Philadelphia, USA

**Keywords:** infection, tha, tka, laboratory, diagnosis, pji

## Abstract

Introduction: Clinical laboratories offer several multipurpose tests, such as the erythrocyte sedimentation rate (ESR) and C-reactive protein (CRP), which are not intended to diagnose any specific disease but are used by clinicians in multiple fields. The results and laboratory interpretation (normal/abnormal) of these multipurpose tests are based on laboratory-reported normal thresholds, which vary across clinical laboratories. In 2018, the International Consensus Meeting on Musculoskeletal Infection (2018 ICM) provided a gold-standard definition to diagnose periprosthetic joint infection (PJI) which included many multipurpose laboratory tests, along with thresholds optimized to diagnose PJI. The discrepancy between laboratory-reported normal thresholds and 2018 ICM-recommended PJI-optimized test thresholds has never been studied. The purpose of this study was to assess the existing variation in laboratory-reported normal thresholds for tests commonly used to diagnose PJI and evaluate the potential diagnostic impact of using laboratory-reported normal thresholds instead of 2018 ICM-recommended PJI-optimized thresholds.

Methods: Clinical laboratories (N=85) were surveyed to determine the laboratory-reported units of measure and normal thresholds for common multipurpose tests to diagnose PJI, including the ESR, CRP, D-dimer, synovial fluid white blood cells (SF-WBC), and polymorphonuclear cell percent (SF-PMN%). The variability of units of measure and normal thresholds for each test was then assessed among the 85 included clinical laboratories. A representative dataset from patients awaiting a revision arthroplasty was used to determine the clinical significance of the existing discrepancy between laboratory-reported normal test interpretations and 2018 ICM-recommended PJI-optimized test interpretations.

Results: Two units of measure for the CRP and six units of measure for the D-dimer were observed, with only 59% of laboratories reporting the CRP in terms of mg/L and only 16% reporting the D-dimer in ng/ml, as needed to utilize the 2018 ICM definition of PJI. Across clinical laboratories surveyed, the mean laboratory-reported normal thresholds for the ESR (20 mm/h), CRP (7.69 mg/L), D-dimer (500 ng/mL), SF-WBC (5 cells/uL), and SF-PMN% (25%) were substantially lower than the 2018 ICM-recommended PJI-optimized thresholds of 30 mm/h, 10 mg/L, 860 ng/mL, 3,000 cells/uL, and 70%, respectively. Interpretation of test results from a representative PJI dataset using each laboratory’s normal test thresholds yielded mean false-positive rates of 14% (ESR), 18% (CRP), 42% (D-dimer), 93% (SF-WBC), and 36% (SF-PMN%) versus the ICM-recommended PJI-optimized thresholds.

Conclusion: When reporting the results for multipurpose laboratory tests, such as the ESR, CRP, D-dimer, SF-WBC, and SF-PMN%, clinical laboratories utilize laboratory-reported units of measure and normal thresholds that are not intended to diagnose PJI, and therefore may not match the 2018 ICM recommendations. Our findings reveal that laboratory-reported normal thresholds for these multipurpose tests are well below the 2018 ICM recommendations to diagnose PJI. Clinical reliance on laboratory-reported results and interpretations, instead of strict use of the 2018 ICM-recommended units and PJI-optimized thresholds, may lead to false-positive interpretation of multipurpose laboratory tests.

## Introduction

Physicians diagnosing periprosthetic joint infection (PJI) have historically had a variety of diagnostic tools at their disposal, with a recent move in the field away from complete reliance on cultures toward a multicriteria definition of PJI. Multicriteria tools such as the 2013 and 2018 International Consensus Meeting (ICM) definitions of PJI require the use of several laboratory tests, many of which are considered multipurpose laboratory tests [1,2]. These multipurpose tests include the erythrocyte sedimentation rate (ESR), C-reactive protein (CRP), D-dimer, synovial fluid white blood cell count (SF-WBC), and polymorphonuclear cell percentage (SF-PMN%). Although laboratories provide their own laboratory-reported normal threshold ranges for each laboratory test, authoritative bodies such as the ICM recommend PJI-optimized thresholds which are intended to override the laboratory-reported normal thresholds for PJI test interpretation. Using these PJI-optimized thresholds, instead of the standard laboratory reference thresholds, is required to appropriately utilize these multiple-criteria diagnostic tools [2].

Laboratories are required to establish a normal reference interval for each test, which is defined as the central 95% of values expected from a healthy local population [3]. This requirement exists to properly account for demographic differences in local populations, such as age, diet, altitude, etc. In many cases, this range can be transferred from the test’s manufacturer with appropriate local verification, while in other cases, a laboratory may create a new normal reference range through testing a minimum of 120 local healthy sample donors [3]. The reference range should not be confused with test result bias, which describes the small differences in results that may be observed at different laboratories analyzing the same samples [3]. For the purposes of this study, it is important to understand that normal reference ranges may vary by laboratory, and the procedures required to set these reference ranges do not involve the diagnosis of PJI and cannot be interpreted and having any meaningful application to the diagnosis of PJI. 

Differences between laboratory-reported normal thresholds and PJI-optimized thresholds may be critically important, as they would produce different sensitivity and specificity performance for each test, and impact the diagnostic accuracy of each test for PJI [4]. Although never studied or quantified in the literature, the use of laboratory-reported normal thresholds by clinicians evaluating a patient for PJI is commonplace in orthopedics. In fact, even recent continuing medical education materials from the American Academy of Orthopaedic Surgeons (AAOS) provide the laboratory-reported normal threshold range for test results in their questions, implying the importance of the laboratory-reported normal threshold in diagnosing PJI [5,6]. Given the ever-changing recommendations of PJI-optimized test thresholds, over time [7,8], between institutions [9,10], and between authoritative bodies and PJI definitions [1,2,11], the widespread adoption and correct use of PJI-optimized laboratory thresholds, instead of laboratory-reported reference thresholds, may not be a realistic expectation. It is reasonable to expect that clinicians unfamiliar with PJI-optimized cutoffs may use laboratory-reported normal thresholds in routine clinical practice for diagnostic decision-making.

Although the complexity of utilizing alternative PJI-optimized test thresholds may be avoided by relying on laboratory-reported normal thresholds, the potential diagnostic impact of this practice has never been evaluated in the literature. The purpose of this study was to assess the existing variation in the laboratory-reported normal thresholds for common PJI tests across a sampling of clinical laboratories and evaluate the potential diagnostic impact of using laboratory-reported normal thresholds instead of ICM-recommended PJI-optimized thresholds.

## Materials and methods

This study was determined to be exempt from institutional review board approval (WCG Institutional Review Board). An earlier version of this study was presented as an abstract on August 7, 2020, at the Musculoskeletal Infection Society Meeting.

Laboratories surveyed

A total of 85 clinical laboratories were contacted by the authors of this study. The sampling of hospitals in this study was determined by the availability of contact at the hospital or laboratory through an author in the study, and an effort to include a combination of both community hospitals and academic centers. This study included laboratories from 18 states in the United States (US), and comprised 41 academic center hospital laboratories and 44 community hospital laboratories, with 85% (70/85) located in the northeast, US. All clinical laboratories in this study were contacted and surveyed by the authors (SF, JD, ZW) to determine standard reporting practices for each test. The following clinical laboratory tests were included in this study: the ESR, CRP, D-dimer, SF-WBC, and SF-PMN%. Both the units of measure and the normal threshold determining the laboratory-reported interpretation for each test were recorded. The number of clinical laboratories that provided a normal threshold for each test was 85 of 85 for ESR, 80 of 85 for CRP, 82 of 85 for d-dimer, 77 of 85 for SF-WBC, and 16 of 85 for SF-PMN%. Some laboratories were unable to provide a normal threshold for all tests because they either do not offer the test or do not report a single normal range associated with the test. 

Data and analysis

The variability in the clinical laboratory-reported units of measure was assessed for each laboratory test across all clinical laboratories. The variability across clinical laboratories of the laboratory-reported normal threshold for test reporting was also assessed. For each test with variations in units across laboratories, all laboratory reference thresholds reported were unit-adjusted using a conversion table to allow for appropriate relative comparisons of laboratory-reported normal thresholds between the 85 laboratories (Table 1). Descriptive statistics were used to describe the range of reference thresholds reported by laboratories in this study.

**Table 1:** Conversion of laboratory reported units to 2018 ICM units ICM: International Consensus Meeting; CRP: C-reactive protein; FEU: fibrinogen equivalent units; DDU: D-dimer units

Test	Laboratory reported units	Conversion factor	2018 ICM unit usage
CRP	mg/L	×1	mg/L
	mg/dL	×10	mg/L
D-dimer	ng/mL FEU	×1	ng/mL
	ug/mL FEU	×1000	ng/mL
	ug/L FEU	×1	ng/mL
	mg/L FEU	×1000	ng/mL
	ng/ml DDU	×2	ng/mL
	mg/L DDU	×2000	ng/mL

The potential clinical impact of the observed variations in laboratory-reported normal test interpretations was then evaluated. We used a representative deidentified clinical dataset of existing laboratory values (ESR, CRP, D-dimer, SF-WBC, SF-PMN%) from previous PJI studies, which is representative of laboratory value distributions observed in the clinical practice of evaluating painful arthroplasties for PJI [12,13]. This clinical dataset of representative raw laboratory values was then subjected to interpretation (normal, high, abnormal, etc) by each laboratory’s normal thresholds, so that each of the 85 laboratories’ standard reference reporting practices was applied to the same representative clinical dataset of laboratory values. The standard diagnostic reporting performance of each clinical laboratory, for each test, was then compared to a gold standard reporting performance that was determined by applying the 2018 ICM-recommended PJI-specific thresholds to the representative clinical dataset (Table 2) [2]. For example, if the laboratory-reported normal threshold reported a CRP of 8 mg/L as “high”, this would be considered a false-positive result compared to the 2018 ICM-recommended PJI-optimized threshold of 10 mg/L. 

**Table 2:** 2018 ICM-recommended PJI-optimized test thresholds for chronic PJI ICM: International Consensus Meeting; ESR: erythrocyte sedimentation rate; CRP: C-reactive protein; SF-WBC: synovial fluid white blood cells; SF-PMN%: synovial fluid polymorphonuclear cell percentage; FEU: fibrinogen equivalent units

Multipurpose tests	PJI-optimized threshold	Units
ESR	30	mm/h
CRP	10	mg/L
D-dimer	860	ng FEU/mL
SF-WBC	3000	cells/uL
SF-PMN%	70	%

The laboratory-reported normal thresholds were generally lower than the 2018 ICM-recommended PJI-optimized threshold, resulting in laboratory-reported interpretations that were false-positive compared to an interpretation using 2018 ICM thresholds. The mean false-positive interpretation rate was calculated for each test across all laboratories and considered clinically significant when greater than 10% of any test’s reported results were categorized as false-positive compared to the ICM standard.

## Results

Units of measure 

The ESR (mm/h), SF-WBC (cell/uL), and SF-PMN% (%) were reported in 2018 ICM-recommended units of measure across all laboratories. In contrast, laboratories variably reported the CRP in a total of two different units of measure (mg/L and mg/dL) and the D-dimer in a total of six different units of measure (ng/mL FEU, ug/mL FEU, ug/L FEU, mg/L FEU, ng/mL DDU, mg/L DDU). Only 59% of laboratories reported the CRP in terms of mg/L and only 16% reported the D-dimer in ng/mL FEU, the respective units required to utilize the 2018 ICM (Table 3).

**Table 3:** Laboratory-reported usage of units of measure ICM: International Consensus Meeting; ESR: erythrocyte sedimentation rate; CRP: C-reactive protein; SF-WBC: synovial fluid white blood cells; SF-PMN%: synovial fluid polymorphonuclear cell percentage; FEU: fibrinogen equivalent units; DDU: D-dimer units

Test	Laboratory-reported units	Clinical laboratory unit usage rate	2018 ICM unit usage
ESR	mm/h	85/85 (100%)	mm/h
CRP	mg/L	47/80 (59%)	mg/L
	mg/dL	33/80 (41%)	mg/L
D-dimer	ng/mL FEU	13/82 (16%)	ng/mL
	ug/mL FEU	32/82 (39%)	ng/mL
	ug/L FEU	2/82 (2%)	ng/mL
	mg/L FEU	20/82 (24%)	ng/mL
	ng/mL DDU	13/82 (16%)	ng/mL
	mg/L DDU	1/82 (1%)	ng/mL
SF-WBC	cells/uL	77/77 (100%)	cells/uL
SF-PMN%	%	16/16 (100%)	%

Laboratory-reported normal test thresholds

The laboratory-reported normal thresholds, to report a test as normal or abnormal to the ordering physician, exhibited variability and differed substantially from the 2018 ICM-recommended PJI-optimized threshold for each test in this study. The median laboratory-reported normal thresholds for the ESR, CRP, D-dimer, SF-WBC, and SF-PMN% across laboratories in this study were 20 mm/h, 7.7 mg/L, 500 ng/mL, 5 cells/uL, and 25%, which are 33%, 23%, 42%, 99%, and 69% lower than the 2018 ICM-recommended PJI-optimized thresholds, respectively (Table 4).

**Table 4:** Descriptive analysis of laboratory-reported normal thresholds versus 2018 ICM-recommended thresholds ICM: International Consensus Meeting; ESR: erythrocyte sedimentation rate; CRP: C-reactive protein; SF-WBC: synovial fluid white blood cells; SF-PMN%: synovial fluid polymorphonuclear cell percentage; FEU: fibrinogen equivalent units

Multipurpose tests	Percent of clinical laboratories reporting standard threshold	Minimum laboratory-reported reference threshold	Maximum laboratory-reported reference threshold	Median laboratory-reported reference threshold	2018 ICM PJI-optimized threshold
ESR (mm/h)	100% (85/85)	10 mm/h	53 mm/h	20 mm/h	30 mm/h
CRP (mg/L)	94% (80/85)	2.9 mg/L	20mg/L	7.7 mg/L	10 mg/L
D-dimer (ng/mL FEU)	96% (82/85)	400 ng/mL FEU	1000 ng/mL FEU	500 ng/mL FEU	860 ng/mL
SF-WBC (cells/uL)	90% (77/85)	0 cells/uL	499 cells/uL	5 cells/uL	3000 cells/uL
SF-PMN%	18% (16/85)	24%	25%	25%	70%

Clinical impact of using laboratory-reported normal test interpretations

Among laboratory results from a cohort of patients being tested for PJI, a substantial proportion of testing results would be laboratory reported as “high/abnormal,” despite being below the 2018 ICM threshold and “negative” for PJI. When compared to the 2018 ICM-recommended PJI-optimized thresholds, laboratory-reported normal thresholds resulted in mean false-positive test rates of 14%,18%, 42%, 93%, and 36% for the ESR, CRP, D-dimer, SF-WBC, and SF-PMN%, respectively, for the diagnosis of PJI across laboratories included in this study. The observed mean false-positive rate of laboratory-reported threshold interpretations relative to 2018 ICM-recommended threshold interpretations was clinically significant (>10%) for all laboratory tests considered (Table 5).

**Table 5:** False-positive rates of utilizing laboratory reported reference thresholds instead of ICM recommended thresholds ICM: International Consensus Meeting; ESR: erythrocyte sedimentation rate; CRP: C-reactive protein; SF-WBC: synovial fluid white blood cells; SF-PMN%: synovial fluid polymorphonuclear cell percentage; FEU: fibrinogen equivalent units

Multipurpose tests	Median laboratory-reported standard threshold	2018 ICM PJI-optimized threshold	Mean laboratory threshold false-positive rate	Minimum laboratory threshold false-positive rate	Maximum laboratory threshold false-positive rate
ESR	20 mm/h	30 mm/h	14% (28/197)	0% (0/197)	37% (73/197)
CRP	7.69 mg/L	10 mg/L	18% (32/176)	0% (0/176)	61% (108/176)
D-dimer	500 ng/mL	860 ng/mL	42% (39/94)	0% (0/94)	65% (61/94)
SF-WBC	5 cells/uL	3000 cells/uL	93% (213/229)	0% (0/229)	100% (228/229)
SF-PMN%	25%	70%	36% (78/216)	36% (78/216)	36% (78/216)

## Discussion

The proper interpretation of laboratory test results is critical for optimal implementation of a multiple-criterion diagnostic tool such as the 2018 ICM definition of PJI. Unfortunately, the laboratory-reported normal test thresholds from clinical laboratories are not intended to diagnose PJI and do not match 2018 ICM recommendations [2]. This study is the first, to our knowledge, to evaluate the discrepancy between the laboratory-reported normal thresholds and ICM-recommended PJI-optimized test thresholds and to assess the potential clinical consequence of clinician reliance on the laboratory-reported normal interpretations of these tests.

This study has identified several pragmatic concerns related to the clinical practice of diagnosing PJI using multiple-criterion tools. First, the CRP and D-dimer, two basic elements of the 2018 ICM definition of PJI, are reported by US clinical laboratories in varying units of measure. In fact, 41% of clinical laboratories reporting CRP results and 84% of clinical laboratories reporting D-dimer results utilize units of measure that differ from the thresholds referenced by the 2018 ICM. The consequence of this reporting difference is the requirement that the ordering clinician converts the result to appropriate units to allow for proper test interpretation using the 2018 ICM-recommended PJI-optimized thresholds. While this conversion may seem simple to those in academic medicine, we believe it likely that many practicing clinicians are failing to make the appropriate conversion in daily clinical practice, and instead rely on the laboratory-reported normal reference test interpretation, leading to suboptimal implementation of the ICM tool. In fact, a recent study in the coronavirus literature expressed a similar concern, having identified existing confusion and errors even in the peer-reviewed literature regarding the reporting and interpretation of D-dimer results [14]. Further research is needed to identify what percentage of clinicians are successfully making the units of conversion, and whether or not this difficulty in unit conversion adversely affects the clinical adoption and performance of multiple-criterion tools by physicians.

Secondly, this study demonstrates a large discrepancy between the laboratory-reported normal test thresholds and those recommended by the 2018 ICM, which is a consequence of the fact that these tests are multipurpose tests that were never designed or optimized by the laboratory to diagnose PJI [2]. Every test assessed in this study, including the ESR, CRP, D-dimer, SF-WBC, and SF-PMN%, had a mean laboratory-reported normal threshold across clinical laboratories that demonstrated a substantial difference from the 2018 ICM recommendations, being set far below the test thresholds that have been recommended as optimal to diagnose PJI by the 2018 ICM [2]. We further demonstrated that a large percentage of laboratory results, from a representative PJI patient sample set, fall into the discrepancy gap between the laboratory-reported normal thresholds and the 2018 ICM-recommended PJI-optimized thresholds for all tests, resulting in the potential for false-positive interpretations if the clinician relies on the laboratory-reported normal test interpretation. Most concerning was the laboratory-reported interpretation of serum CRP results, a universally utilized test for PJI, which demonstrated a mean 18% false-positive “high or abnormal” interpretation rate when compared to the ICM-recommended PJI-optimized interpretation. A false-positive interpretation of laboratory values could move a patient into the infected category of the 2018 ICM PJI diagnostic criteria, biasing a surgeon inappropriately toward surgical options and prolonged antibiotics that may not be necessary.

This study’s finding, that there is a clinically important discordance between laboratory-reported test interpretations and those recommended by the 2018 ICM to diagnose PJI, suggests that it is important and impactful for clinicians to ignore the laboratory-reported normal test interpretation from the clinical laboratory, and instead convert to appropriate units of measure and apply the 2018 ICM-recommended PJI-optimized threshold. While using the 2018 ICM-recommended PJI-optimized threshold for a multipurpose or off-label test may seem a simple task to academic surgeons who perform PJI research, it exists as a potential barrier to widespread appropriate clinical adoption of the 2018 ICM tool and a source of potential error during its clinical use [15,16]. Complexity and the need to execute multiple rules are known barriers to guideline compliance among clinicians, as tasks that seem straightforward to experienced clinicians may cause confusion among less experienced clinicians [16,17]. While there is little PJI research assessing the appropriate implementation of multiple-criterion diagnostic tools by practicing physicians, a recent study demonstrates poor inter-physician agreement even when physicians are directed to use a specific multiple-criterion tool to diagnose PJI [18]. Our study’s findings of discordance between laboratory-reported and ICM-recommended test interpretations offer one rationale explaining inconsistency in implementation of a multiple-criterion tool to diagnose PJI.

Limitations

This study only involves laboratories in the US but did not include all US laboratories. This weakness is mitigated by the fact that 85 laboratories were included, and a relatively even distribution of academic and community hospitals was chosen to provide some diversity of laboratories across 18 states. It is always possible that the subset of laboratories chosen introduced bias, although the observed variabilities in testing units and laboratory-reported normal thresholds would still remain concerning. Additionally, the presumption of this study is that some clinicians utilize laboratory-reported normal thresholds and interpretations instead of 2018 ICM recommended thresholds. Although this presumption is not supported by any existing literature, as there has never been a study evaluating this specific practice, the authors of this study believe that this presumption is reasonably obvious in daily practice, as colleagues routinely use laboratory-reported results (normal/abnormal) in decision-making to diagnose PJI. This is especially observable in routine practice interpreting CRP and D-dimer results, which have various units of measure and laboratory-reported normal thresholds across laboratories.

## Conclusions

The first conclusion of this study is that clinical laboratories exhibit variable reporting of both the units and standard thresholds for the multipurpose tests used to diagnose PJI. Although laboratories demonstrated consistency in the units used to report the results of the ESR (mm/h), SF-WBC (cells/uL), and SF-PMN% (%), variability in units usage was observed in the reporting of the CRP and D-dimer result, with D-dimer results reported in a total of six different units of measure. In addition to reporting variable units of measure, laboratories exhibited variable normal thresholds utilized to report a test as normal versus abnormal. In general, clinical laboratories used normal reporting thresholds that were below the 2018 ICM-recommended thresholds to optimally diagnose PJI.

The second conclusion of this study is that the differences between laboratory-reported normal thresholds and 2018 ICM-recommended thresholds for PJI are clinically significant, as clinician usage of the standard laboratory interpretation, instead of the ICM-recommended interpretation, would have resulted in greater than a 10% false-positive interpretation rate for all laboratory tests studied. Therefore, clinicians who utilize laboratory-reported results (normal/abnormal), instead of applying the 2018 ICM recommendations, are at risk of misinterpreting the tests routinely used to diagnose PJI.
